# Supplementary material for: Active site specificity profiling datasets of matrix metalloproteinases (MMPs) 1, 2, 3, 7, 8, 9, 12, 13 and 14
Source: Data Brief. 2016 Feb 22;7:299–310. doi: 10.1016/j.dib.2016.02.036 (PMC4777984; doi:10.1016/j.dib.2016.02.036)
Supplement: Supplementary file 10 — Supplementary material [file mmc10.zip › WebPICS_hMMP9_T_1%/P2.html]

 

PICS results


|  |  |
| --- | --- |
| **P2\_C**  2 in 138 sites   1.4 %    effects > 10 perc. pnts.  (vice-versa in brackets)  P2prime\_C: 46.4 (18.6) |  |
| **P2\_F**  6 in 138 sites   4.3 %    effects > 10 perc. pnts.  (vice-versa in brackets)  P1\_P: 24.6 (12.4)   P1prime\_Q: 26.8 (17.9)   P1prime\_V: 41.3 (20.7)   P2prime\_I: 39.1 (15.7)   P3prime\_H: 12.4 (12.4)   P3prime\_N: 28.2 (24.3) |  |
| **P2\_G**  11 in 138 sites   8.0 %    effects > 10 perc. pnts.  (vice-versa in brackets)  P1\_E: 11.0 (12.0)   P1\_Q: 11.7 (14.2) |  |
| **P2\_H**  6 in 138 sites   4.3 %    effects > 10 perc. pnts.  (vice-versa in brackets)  P1\_W: 14.5 (29.0)   P1prime\_Q: 26.8 (17.9)   P2prime\_F: 12.4 (12.4)   P3prime\_H: 12.4 (12.4) |  |
| **P2\_M**  9 in 138 sites   6.5 %    effects > 10 perc. pnts.  (vice-versa in brackets)  P1prime\_V: 24.6 (18.5)   P2prime\_M: 27.5 (31.0)   P3prime\_N: 17.1 (22.1) |  |
| **P2\_Q**  8 in 138 sites   5.8 %    effects > 10 perc. pnts.  (vice-versa in brackets)  P1\_N: 17.8 (14.2)   P2prime\_V: 25.9 (13.0) |  |
| **P2\_S**  18 in 138 sites   13.0 %    effects > 10 perc. pnts.  (vice-versa in brackets)  P1\_S: 12.1 (15.6) |  |
| **P2\_V**  10 in 138 sites   7.2 %    effects > 10 perc. pnts.  (vice-versa in brackets)  P1\_D: 23.5 (26.1)   P1prime\_C: 16.4 (32.8)   P2prime\_M: 14.2 (17.8) |  |
| **P2\_Y**  7 in 138 sites   5.1 %    effects > 10 perc. pnts.  (vice-versa in brackets)  P1\_H: 11.4 (19.9)   P1\_N: 21.4 (14.9)   P1\_S: 32.8 (16.3)   P1prime\_C: 10.7 (14.9) |  |
